# Corticosteroids reduce pathologic interferon responses by downregulating STAT1 in patients with high-risk COVID-19

**DOI:** 10.1038/s12276-023-00964-8

**Published:** 2023-03-20

**Authors:** Hyun-Woo Jeong, Jeong Seok Lee, Jae-Hoon Ko, Seunghee Hong, Sang Taek Oh, Seongkyun Choi, Kyong Ran Peck, Ji Hun Yang, Seok Chung, Sung-Han Kim, Yeon-Sook Kim, Eui-Cheol Shin

**Affiliations:** 1 Department of Tissue Morphogenesis, Max Planck Institute for Molecular Biomedicine, Münster, 48149 Germany; 2 Graduate School of Medical Science and Engineering, Korea Advanced Institute of Science and Technology (KAIST), Daejeon, 34141 Republic of Korea; 3 Division of Infectious Diseases, Department of Medicine, Samsung Medical Center, Sungkyunkwan University School of Medicine, Seoul, 06351 Republic of Korea; 4 Department of Biochemistry, College of Life Science and Biotechnology, Yonsei University, Seoul, 03722 Korea; 5 School of Mechanical Engineering, Korea University, Seoul, 02841 Republic of Korea; 6 KU-KIST Graduate School of Converging Science and Technology, Korea University, Seoul, 02841 Republic of Korea; 7 Department of Infectious Diseases, Asan Medical Center, University of Ulsan College of Medicine, Seoul, 05505 Republic of Korea; 8 Division of Infectious Diseases, Department of Internal Medicine, Chungnam National University School of Medicine, Daejeon, 35015 Republic of Korea; 9 The Center for Viral Immunology, Korea Virus Research Institute, Institute for Basic Science (IBS), Daejeon, 34126 Republic of Korea

**Keywords:** Viral infection, Monocytes and macrophages

## Abstract

We do not yet understand exactly how corticosteroids attenuate hyperinflammatory responses and alleviate high-risk coronavirus disease 2019 (COVID-19). We aimed to reveal the molecular mechanisms of hyperinflammation in COVID-19 and the anti-inflammatory effects of corticosteroids in patients with high-risk COVID-19. We performed single-cell RNA sequencing of peripheral blood mononuclear cells (PBMCs) from three independent COVID-19 cohorts: cohort 1 was used for comparative analysis of high-risk and low-risk COVID-19 (47 PBMC samples from 28 patients), cohort 2 for longitudinal analysis during COVID-19 (57 PBMC samples from 15 patients), and cohort 3 for investigating the effects of corticosteroid treatment in patients with high-risk COVID-19 (55 PBMC samples from 13 patients). PBMC samples from healthy donors (12 PBMC samples from 12 donors) were also included. Cohort 1 revealed a significant increase in the proportion of monocytes expressing the long noncoding RNAs *NEAT1* and *MALAT1* in high-risk patients. Cohort 2 showed that genes encoding inflammatory chemokines and their receptors were upregulated during aggravation, whereas genes related to angiogenesis were upregulated during improvement. Cohort 3 demonstrated downregulation of interferon-stimulated genes (ISGs), including STAT1, in monocytes after corticosteroid treatment. In particular, unphosphorylated STAT-dependent ISGs enriched in monocytes from lupus patients were selectively downregulated by corticosteroid treatment in patients with high-risk COVID-19. Corticosteroid treatment suppresses pathologic interferon responses in monocytes by downregulating STAT1 in patients with high-risk COVID-19. Our study provides insights into the mechanisms underlying COVID-19 aggravation and improvement and the effects of corticosteroid treatment.

## COVID-19: Corticosteroids counteract inflammation by interferon

The ability of corticosteroid drugs to alleviate extreme inflammation in severe cases of COVID-19 is linked to their reduction of the gene expression that is stimulated by interferon, an immunological cytokine protein. Interferon is crucial for a healthy immune response, but when produced in excess it can play a central role in causing damaging inflammation. Hyun-Woo Jeong at the Max Planck Institute for Molecular Biomedicine, Münster, Germany, and colleagues in South Korea compared the activity of genes involved in the inflammatory response in blood cells from COVID-19 patients and healthy donors. The results provided insights into the role of altered gene activity in both aggravation and improvement of COVID-19. Of particular interest was the ability of corticosteroids to downregulate the activity of a gene called *STAT1*, involved in mediating interferon signals.

## Introduction

Coronavirus disease 2019 (COVID-19), a current pandemic disease, is caused by infection with severe acute respiratory syndrome coronavirus 2 (SARS-CoV-2), a newly emerging virus^1^. SARS-CoV-2 infection clinically manifests in diverse forms, from asymptomatic infection to severe/critical COVID-19 accompanied by acute respiratory distress syndrome and multiorgan failure^2^. Children and healthy young adults tend to have asymptomatic infection or mild COVID-19 upon SARS-CoV-2 infection^3^. However, SARS-CoV-2 infection can progress to severe/critical COVID-19 at a high rate among elderly individuals and individuals with underlying chronic illness^4^.

Since the emergence of COVID-19, many studies have been performed to understand the pathophysiological mechanisms underlying severe/critical COVID-19 and have shown that hyperinflammatory responses are at the center of the progression to severe/critical COVID-19^5,6^. The hyperinflammatory responses in patients with severe/critical COVID-19 are attributed to exaggerated activation of myeloid cells, including neutrophils and monocytes/macrophages, and overproduction of proinflammatory cytokines^7,8^. In patients with severe/critical COVID-19, increased serum or plasma levels of tumor necrosis factor (TNF), interleukin (IL)-6, IL-1β, and other cytokines and chemokines have been observed^9^. In addition, dysregulated type I interferon (IFN) responses and aberrant activation of complement pathways have been reported in patients with severe/critical COVID-19^10,11^.

The hyperinflammatory responses in patients with severe/critical COVID-19 have been investigated by single-cell RNA sequencing (scRNA-seq) of peripheral blood mononuclear cells (PBMCs) or bronchoalveolar lavage (BAL) fluid^12–15^. Myeloid cells, such as CD14^+^ monocytes and granulocytes, undergo profound changes in patients with severe/critical COVID-19. For example, the levels of dysfunctional HLA-DR^lo^CD163^hi^ and HLA-DR^lo^S100A^hi^CD14^+^ monocytes are typically increased in patients with severe COVID-19^12^. In addition, immature and dysfunctional neutrophil populations are increased in patients with severe COVID-19, indicating inflammation-associated myelopoiesis^14^. Moreover, severe COVID-19 is characterized by the coexistence of type I IFN responses and TNF/IL-1β–driven inflammatory features^16^.

Type I IFNs play paradoxical roles in COVID-19^11^. A robust early type I IFN response leads to early viral clearance, preventing progression to severe COVID-19^17^. Patients with type I IFN deficiency due to inborn errors or type I IFN-specific autoantibodies suffer from severe/critical COVID-19 because of uncontrolled viral replication^18,19^. However, delayed and exaggerated type I IFN responses contribute to the aggravation of host-damaging inflammatory responses rather than viral control, leading to the progression to severe COVID-19^20^. Given the complicated effects of type I IFNs in COVID-19, there is a need to examine the type I IFN signaling pathways, responsive genes, and their roles in detail during the natural course of COVID-19.

The current standard of care for patients with severe/critical COVID-19 is corticosteroid treatment^21^. Corticosteroids are used for the treatment of chronic inflammatory and autoimmune diseases because of their well-known anti-inflammatory effects^22,23^. However, the exact anti-inflammatory mechanisms of corticosteroids have not been elucidated, although corticosteroids are known to exert their actions by binding to intracellular steroid receptors^24,25^. Corticosteroid treatment is often accompanied by immunosuppression-related side effects that can be detrimental in patients with infectious diseases^26,27^. Understanding the anti-inflammatory mechanisms of corticosteroids in severe/critical COVID-19 will enable us to develop alternative anti-inflammatory therapeutics that do not suppress beneficial antiviral immune responses.

In the present study, we performed scRNA-seq analysis of PBMCs from three independent COVID-19 cohorts: cohort 1 was used for comparative analysis of high-risk and low-risk COVID-19, cohort 2 for longitudinal analysis during the course of COVID-19, and cohort 3 for investigating the effects of corticosteroid treatment in patients with high-risk COVID-19. The current study provides insights that improve the understanding of the mechanisms underlying the progression to severe COVID-19 or spontaneous resolution of COVID-19 and the effects of corticosteroid treatment. This information will contribute to improving the clinical management of patients with high-risk COVID-19.

## Methods

### Patients

Patients diagnosed with COVID-19 and healthy volunteers were enrolled from Chungnam National University Hospital, Asan Medical Center, and Samsung Medical Center. SARS-CoV-2 RNA was detected in patients’ nasopharyngeal swab and sputum specimens by multiplex real-time reverse-transcriptase PCR using an Allplex™ 2019-nCoV Assay kit (Seegene, Seoul, Republic of Korea). Patients’ clinical features, laboratory findings, and chest radiographs were collected from their electronic medical records at each hospital. PBMCs were collected from healthy volunteers before 2019, prior to the COVID-19 pandemic, and the healthy volunteers had no history of taking corticosteroids because all samples were collected during regular health checkups with a detailed medical history. The study protocol was reviewed and approved by the institutional review boards of all participating institutions. Written informed consent was obtained from all patients.

### PBMC isolation and preservation

Peripheral blood mononuclear cells (PBMCs) were isolated from whole blood by standard Ficoll-Paque (GE Healthcare, Uppsala, Sweden) density gradient centrifugation, frozen in freezing medium, and stored in a liquid nitrogen tank until use.

### Flow cytometry and immunophenotyping

PBMCs were stained with fluorochrome-conjugated antibodies against surface markers for 20 min on ice and then washed. Dead cells were excluded using a Live/Dead fixable cell stain kit (Invitrogen, Carlsbad, CA). For intracellular staining, surface-stained cells were permeabilized using a Foxp3 staining buffer kit (eBioscience, San Diego, CA) according to the manufacturer’s instructions and further stained for intracellular proteins. Flow cytometry was performed on an LSR II instrument using FACSDiva software (BD Biosciences), and the data were analyzed using FlowJo software (TreeStar, San Carlos, CA).

### Single-cell RNA-seq library preparation

Freshly frozen PBMCs were thawed in a water bath at 37 °C for 1 min and transferred to a 50 ml tube, and then 10 volumes of warm RPMI 1640 medium supplemented with 10% FCS and 0.1 mg/ml DNase I were added slowly, followed by centrifugation at 300 × *g* for 5 min. The cell pellet was collected, and the cell number and viability were determined using trypan blue staining evaluated on a Luna-II automated cell counter (Logos Biosystems). Each PBMC sample was labeled with an appropriate sample multiplexing antibody (Single-Cell multiplex kit - Human; BD Biosciences), and then up to six samples were pooled together in equal numbers of 10,000 cells (60,000 cells in total) and loaded on a microwell cartridge of the BD Rhapsody Express system (BD Biosciences). Single-cell whole transcriptome analysis libraries were prepared according to the manufacturer’s instructions using a BD Rhapsody WTA Reagent kit (BD, 633802) and sequenced on an Illumina HiSeq X using 2 × 75 bp paired-end reads with an 8-bp single index.

### Single-cell RNA-seq data processing

FASTQ format raw sequencing data were processed in the BD Rhapsody WTA Analysis Pipeline (version 1.0) on a SevenBridges Genomics online platform (SevenBridges) using a human reference genome (GRCh38) for alignment and respective sample multiplexing tag IDs for sample demultiplexing. Gene × cell expression matrices of 171 total samples were extracted using the RSEC_Adjusted_Molecules values. Data normalization and further analysis were performed using Seurat (version 3.1.5) unless otherwise specified. For initial quality control of the extracted gene-cell matrices, we filtered cells with the parameters nFeature_RNA > 500 & nFeature_RNA < 3000 for the number of genes per cell, percent.mito < 25 for the percentage of mitochondrial genes, and genes with parameter min.cell = 3. The filtered matrices were normalized using LogNormalize with a scale factor of 10,000. Variable genes were identified with the parameters selection.method = vst, mean.function = ExpMean, dispersion.function = LogVMR, x.low.cutoff = 0.0125, x.high.cutoff = 3, and y.cutoff = 0.5. Reciprocal PCA was used for the integration of all sample datasets. Features that were repeatedly variable across the datasets were selected using the SelectIntegrationFeatures function and used for the PCA on each dataset, and then the FindIntegrationAnchors and IntegrateData functions with default options were used for data integration. Significant principal components were determined by the JackStraw method, and the top 11 principal components were selected for downstream analysis by UMAP nonlinear dimensionality reduction and unsupervised hierarchical clustering analysis (resolution 0.2). The cellular identity of each cluster was determined by finding cluster-specific marker genes using the FindAllMarkers function with a minimum fraction of cells expressing the gene over 25% (min.pct = 0.25) and comparing those markers to known cell type-specific genes identified in previous studies and further confirmed using the R package SingleR, which compares the transcriptome of each single cell to reference datasets to determine cellular identity.

### Gene Ontology term enrichment analysis

Differentially expressed genes or cluster-specific marker genes were identified using the FindMarkers or FindAllMarkers functions of the Seurat package, respectively. Enriched Gene Ontology terms in the molecular function, biological function, and cellular component categories were identified using the enrichGO function of the clusterProfiler R package with a q value cutoff of 0.1 and then simplified by removing redundant enrichment terms via the simplify function with a cutoff of 0.7, by = p.adjust, and measure = Wang options. The top GO terms for each condition or sample were selected by adjusted *P* values.

### Cell–cell interactome analysis

The R package iTALK (10.1101/507871) was used for ligand–receptor interactome analysis. Differentially expressed genes during disease aggravation or improvement were identified in each cell type using the nonparametric Wilcoxon rank-sum test and used for the detection of ligand–receptor pairs using the FindLR function. The results were plotted using the LRPlot function with datatype = DEG.

### Gene set enrichment analysis using Enrichr and FGSEA

Enrichr, web-based software for gene set enrichment analysis, was used to analyze HALLMARK pathways^28^, and the log(P value) was calculated for enriched pathways with an adjusted *P* value < 0.05. Gene set enrichment analysis was performed using the Molecular Signatures Database (MSigDB) v7.1 KEGG subset of canonical pathways with the fgsea R package (Korotkevich, G., Sukhov, V. & Sergushichev, A. Fast gene-set enrichment analysis. Preprint at BioRxiv 10.1101/060012 (2019)). Differentially expressed genes between different groups were identified using the FindMarkers function in Seurat with min.pct = 0.25 and logfc.threshold = 0, preranked according to the average log2-fold change values and used for enriched gene set identification using the gfsea function with minSize = 15, maxSize = 500, and nperm = 1000.

### SLE PBMC single-cell RNA-seq monocyte cluster analysis

A single-cell RNA-seq dataset for PBMCs from SLE and healthy donors was obtained from GEO (GSE135779). This dataset included ~276k single PBMCs obtained from 33 SLE and 11 healthy matched donors. After multiplet removal using scrublet, matrices were analyzed using the Python-based Scanpy workflow (https://scanpy.readthedocs.io/en/stable/), which includes preprocessing, visualization, and clustering. The overall analysis pipeline and parameter settings were similar to what the original authors used as available at github.com/dnehar/SingleCells_SLE_paper. Among monocytes, the ‘score_genes’ function in Scanpy was used to calculate *p*-ISG and u-ISG scores.

## Results

### scRNA-seq analysis of PBMCs from three independent COVID-19 cohorts

**Fig. 1:**
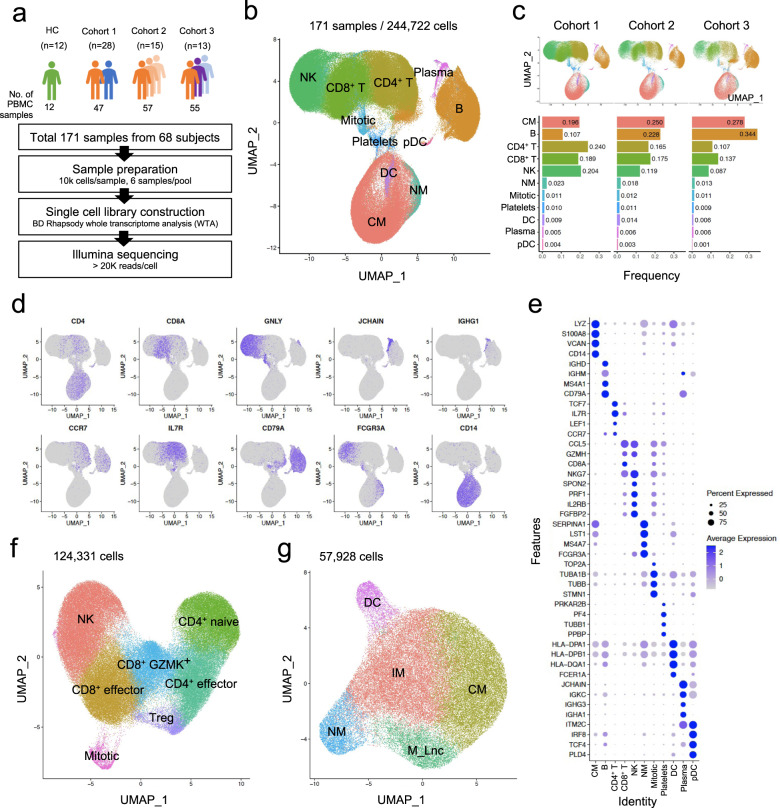
Single-cell transcriptomics of PBMCs from COVID-19 patients. **a** Overall cohort design and analysis. **b** UMAP plot of PBMCs from all three cohorts. **c** Cell type frequency in each cohort. **d** UMAP plots depicting the expression of representative marker genes in each cell type. Colors represent the relative gene expression. **e** Dot plot showing the top four markers for each cell type. Dot size indicates the percentage of cells in which the gene was detected (“Percent Expressed”). Colors demonstrate the average expression level of the gene (scaled data) in each cluster (“Average Expression”). **f** UMAP plots showing subclusters of T- and NK-cell and (**g**) myeloid populations.

In the present study, three independent COVID-19 cohorts were enrolled for scRNA-seq analysis of PBMCs (Fig. 1a and Supplementary Table 1). Cohort 1 was designed for comparative analysis of high-risk and low-risk COVID-19 (47 PBMC samples from 28 patients). PBMC specimens were designated as high-risk or low-risk COVID-19 according to the National Early Warning Score (NEWS), a clinical criterion used to discriminate patients at risk of early cardiac arrest, unanticipated intensive care unit admission, and death^29^. High risk (NEWS ≥ 7) or low risk (NEWS ≤ 4) was determined on the day of blood sampling^29^. Demographic information is provided with detailed clinical information in Supplementary Table 1. Cohort 2 was designed for longitudinal analysis during the course of COVID-19, including aggravating and improving disease courses (57 PBMC samples from 15 patients). Cohort 3 was designed to analyze the effects of corticosteroid treatment (55 PBMC samples from 13 patients). PBMC samples from healthy donors (12 PBMC samples from 12 donors) were also included. PBMCs were freshly frozen and thawed immediately before preparing a single-cell library with the BD Rhapsody workflow. Six samples were pooled for each round of experiments using a BD human sample multiplexing kit. After checking library quality, sequencing was performed on an Illumina platform with an estimated depth of 20,000 and 200 reads per cell for transcriptome and sample tags, respectively. The raw sequencing data were processed by the BD Rhapsody WTA pipeline, and then we performed in-depth bioinformatic analysis (Supplementary Fig. 1a).

A UMAP plot shows the overall cellular landscape of 244,722 cells from a total of 171 PBMC samples in the three cohorts. We identified 11 major cell types, namely, NK cells, CD8^+^ T cells, CD4^+^ T cells, B cells, plasma cells, classical monocytes (CMs), nonclassical monocytes (NMs), dendritic cells (DCs), plasmacytoid DCs (pDCs), mitotic cells, and platelets (Fig. 1b). The relative proportions of the identified cell types for each cohort are shown in Fig. 1c. The cell type annotation was fully consistent with cell type-specific markers and known gene expression patterns, as demonstrated in the UMAP feature plots (Fig. 1d), dot plots (Fig. 1e), and violin plots (Supplementary Fig. 1b) depicting the expression of each gene.

Next, we performed subclustering analyses of the two major populations, the T/NK-cell and myeloid populations. The T/NK-cell population was subclustered into NK cells, effector CD8^+^ T cells, GZMK^+^ CD8^+^ T cells, effector CD4^+^ T cells, naïve CD4^+^ T cells, and regulatory T cells (Fig. 1f, Supplementary Fig. 1c, and Supplementary Table 2). CMs, intermediate monocytes (IMs), NMs, and DCs were identified in the myeloid population (Fig. 1g, Supplementary Fig. 1d,and Supplementary Table 2). In addition, a subcluster of monocytes characterized by high expression of the long noncoding RNAs *NEAT1* and *MALAT1* was identified and named ‘monocyte_Lnc’.

### Comparative analysis of high-risk and low-risk COVID-19

Cohort 1 included high-risk (31 PBMC samples from 12 patients) and low-risk (16 PBMC samples from 16 patients) COVID-19 patients (Supplementary Fig. 2a). We analyzed differentially expressed genes (DEGs) for each cell type by comparing the healthy control, low-risk COVID-19 patient, and high-risk COVID-19 patient groups. We summarized the significantly enriched Gene Ontology terms in each cell type for DEGs between the patients with high-risk and low-risk COVID-19 (Supplementary Fig. 2b). The low-risk group had more enriched gene expression for ‘cellular response to interferon-gamma’ in CMs, ‘antigen receptor-mediated signaling pathway’ in B cells and CMs, and ‘antigen processing and presentation of exogenous antigen’ in CMs, CD4^+^ T cells, DCs, and pDCs. The high-risk group had more enriched gene expression for ‘myeloid cell differentiation’ in DCs, ‘granulocyte activation’ in CMs and NMs, and ‘cellular response to type I interferon’ in B cells, CD4^+^ T cells, and NK cells.

As described above, the monocyte_Lnc population expressing high levels of *NEAT1* and *MALAT1* subclustered within the myeloid population (Supplementary Fig. 3a). The proportion of the monocyte_Lnc subcluster was significantly increased in high-risk patients compared to healthy controls or low-risk patients (Supplementary Fig. 3b; *p* = 0.003). Moreover, the transcript levels of *NEAT1* and *MALAT1* in the monocyte_Lnc subcluster were increased in high-risk disease (Supplementary Fig. 3c). By DEG analysis, we confirmed the upregulation of *NEAT1* and *MALAT1* in the monocyte_Lnc population compared to CMs/IMs (Supplementary Fig. 3d). Genes associated with ‘TNF-α signaling via NF-κB’, ‘inflammatory response’, ‘IFN-γ response’, ‘IL-6/JAK/STAT3 signaling’, and ‘IFN-α response’ were significantly enriched among the upregulated genes in the monocyte_Lnc subcluster (Supplementary Fig. 3e). Taken together, the findings indicate that the monocyte_Lnc subcluster that was significantly increased in high-risk COVID-19 comprises highly inflammatory cells stimulated by multiple cytokines, although the roles of *NEAT1* and *MALAT1* in the regulation of these inflammatory cells remain to be elucidated.

### Longitudinal analysis during the course of COVID-19

Next, we examined molecular changes in peripheral blood immune cells during the course of COVID-19 by analyzing the longitudinal data of cohort 2. In this cohort, 4 pairs were analyzed during disease aggravation, and 14 pairs were analyzed during disease improvement (Supplementary Fig. 4a). Aggravation and improvement were determined on the basis of the change in the NEWS, which was concordant with radiological changes and C-reactive protein levels (Supplementary Fig. 4b–d).

**Fig. 2:**
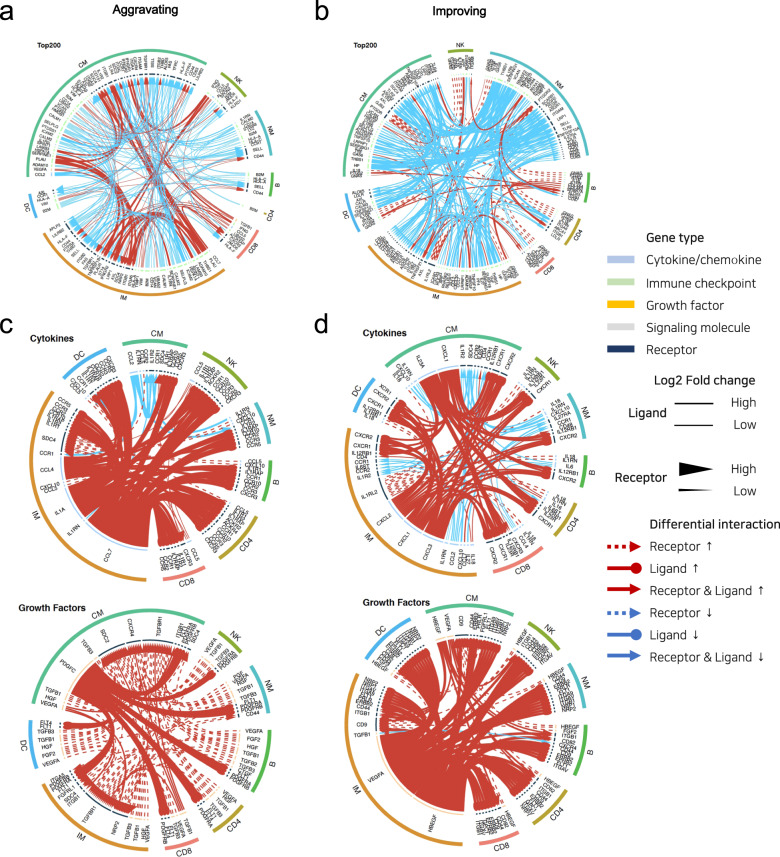
Crosstalk between immune cells characterizes aggravation and improvement of disease. **a**, **c** Interactome analysis of potential interactions between different immune cell types during the aggravation and **b**, **d** improvement phases. The widths of lines and arrows represent a relative log2-fold change in ligands and receptors, respectively. Dotted lines with an arrowhead represent differential levels of a receptor; solid lines with a dot represent differential levels of a ligand; solid lines with an arrowhead represent simultaneous differential levels of a ligand and receptor. Red indicates upregulated gene expression; cyan indicates downregulated gene expression.

To understand the mechanisms underlying disease aggravation and improvement, we focused on comprehensive cell-to-cell interactomes among multiple cell types. During aggravation, CMs and IMs showed the most significant and variable changes in signaling communication (Fig. 2a). CMs and IMs highly interact with multiple cell types. In contrast, during improvement, downmodulation of cell-to-cell interactions was observed regardless of cell type (Fig. 2b). In particular, multiple interactions were modulated in NMs, which are known to be involved in the resolution of inflammation.

We then focused on cytokines and growth factors. During aggravation, chemokines that are known to recruit immune cells to peripheral inflammatory sites and their receptors were upregulated in pairs in multiple cell types, including CCL4–CCR5, CCL5–CCR1/CCR3/CCR5, and CXCL10–CXCR3 (Fig. 2c, upper). More importantly, IL-1-related molecules, including IL1A, IL1R1, IL1R2, and IL1RAP, were upregulated in multiple cell types, particularly in CMs and IMs. These data show that inflammatory cell migration accompanied by the activation of IL-1 pathways is involved in the aggravation of COVID-19. Among growth factors, interactions between PDGFC, which is related to vascular inflammation, and its receptors were predominantly observed (Fig. 2c, lower).

During improvement, IL-23A expression was increased in CMs and that of its receptor was increased in CD8^+^ and CD4^+^ T cells. Interestingly, during disease improvement, chemokines that are known to be involved in angiogenesis and their receptors were upregulated in pairs in multiple cell types, including CXCL1/CXCL2/CXCL3–CXCR2 (Fig. 2d, upper). In addition, the upregulation of VEGFA and HBEGF, which are known to play critical roles in angiogenesis and wound healing, was predominantly observed in CMs and IMs during disease improvement (Fig. 2d, lower), as reported previously^30,31^.

Thus, enhanced inflammatory responses characterized by inflammatory chemokines and IL-1 were observed during disease aggravation, whereas tissue regeneration responses characterized by angiogenic/wound healing growth factors, and chemokines became prominent during disease improvement.

### Effects of corticosteroid treatment

**Fig. 3:**
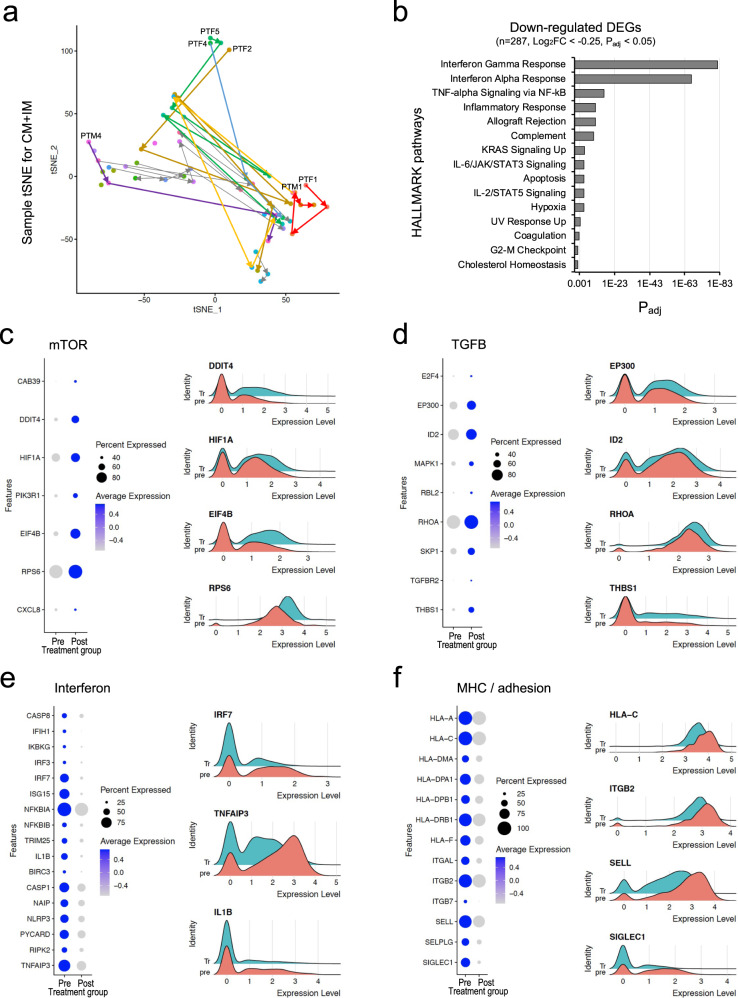
Corticosteroid treatment suppresses the type-I IFN response in classical monocytes (CMs) and intermediate monocytes (IMs). **a** tSNE plot of pseudobulk gene expression values for CMs/IMs in each sample. Colors represent individual patients. Arrows indicate the progression of time. Six patients with a pretreatment sample are labeled with text. **b** HALLMARK pathway enrichment analysis of genes downregulated by corticosteroid treatment. Padj, adjusted *P* value. **c**–**f** Expression of genes related to key signaling pathways. Left, dot plots depicting the percentage of cells in which the gene was detected (“Percent Expressed”) and average expression level of the gene (scaled data) in each cluster (blue, “Average Expression”). Right, ridge plots depicting the expression of genes changed between pre- and post-corticosteroid treatment.

Finally, we analyzed cohort 3 (55 PBMC samples from 13 patients) to identify the molecular effects of corticosteroids, which are approved for the treatment of patients with high-risk COVID-19 (Supplementary Fig. 5a, b). First, we performed dimensionality reduction using the average gene expression values of CMs/IMs for each sample to visualize the sample-to-sample proximities and reveal the phenotypic heterogeneity of corticosteroid responses (Fig. 3a and Supplementary Fig. 5a). Pretreatment PBMC samples were obtained from six patients (PTF1, PTF2, PTF4, PTF5, PTM1, and PTM4). Interestingly, the pretreatment samples from three patients (PTF2, PTF4, and PTF5) clustered together, indicating that a hyperinflammatory status was shared by patients with high-risk COVID-19. However, the changes following corticosteroid treatment were heterogeneous among the individual patients.

In further analyses, we focused on paired PBMC samples obtained from the six patients before and 1 day after starting corticosteroid treatment. We found that among several immune cell types, DEGs were most abundant in CMs/IMs (Supplementary Fig. 5c). We performed gene set enrichment analysis with Hallmark pathways^28^ using the DEGs of CMs/IMs from the six paired samples. Several pathways were significantly downregulated by corticosteroid treatment, including ‘IFN-γ response’, ‘IFN-α response’, ‘TNF-α signaling via NF-κB’, ‘allograft rejection pathway’, ‘complement pathway’, and ‘inflammatory response’ (Fig. 3b).

We found that the mTOR signaling pathway was upregulated in CMs/IMs obtained 1 day after corticosteroid treatment (Fig. 3c). In particular, we found increased levels of several target genes related to mTOR pathways, such as HIF1A, EIF4B, and RPS6, and some upstream regulators, including PIK3R1 and DDIT4. Notably, mTOR signaling activity is required for the anti-inflammatory effects of corticosteroids^32^. Therefore, the upregulation of mTOR pathways may contribute to the anti-inflammatory effects of corticosteroids in patients with high-risk COVID-19. In addition, the TGFB signaling pathway was upregulated in CMs/IMs obtained 1 day after corticosteroid treatment (Fig. 3d). Considering that TGF-β suppresses the inflammatory activity of diverse immune cells and contributes to tissue regeneration and wound healing, the current data suggest that corticosteroid treatment plays dual roles, namely, anti-inflammatory and tissue-regenerative functions, in patients with severe COVID-19. When we analyzed the frequency of the monocyte_Lnc subcluster, we found no significant difference between pre- and post-corticosteroid treatment (Supplementary Fig. 5d).

We further analyzed genes downregulated in CMs/IMs obtained 1 day after corticosteroid treatment. We found that signaling pathways related to type I IFNs and MHC/adhesion were typically downregulated after corticosteroid treatment (Fig. 3e, f). By flow cytometric analysis, we confirmed that the expression of HLA-DR, a human MHC class II protein, was significantly reduced on CD14^+^CD16^-^ classical monocytes after corticosteroid treatment (Supplementary Fig. 5e, f).

Previous reports have demonstrated that type I IFN responses are delayed but exaggerated in patients with severe COVID-19^20,33^. Moreover, exaggerated type I IFN responses aggravate TNF/IL-1-driven inflammatory responses in severe COVID-19^16^. Given the inflammatory role of type I IFN responses, the current findings suggest that corticosteroids exert anti-inflammatory effects by downregulating IFN pathways. Previous reports have also shown that genes related to MHC and antigen presentation are downregulated in patients with severe/critical COVID-19^5^ and, consequently, antigen presentation to T cells is impaired^34^. It is anticipated that corticosteroid treatment may further decrease antigen presentation, leading to severely impaired T-cell responses, although hyperinflammatory responses are also suppressed by corticosteroids.

### Suppression of prolonged type I IFN responses by corticosteroid treatment

**Fig. 4:**
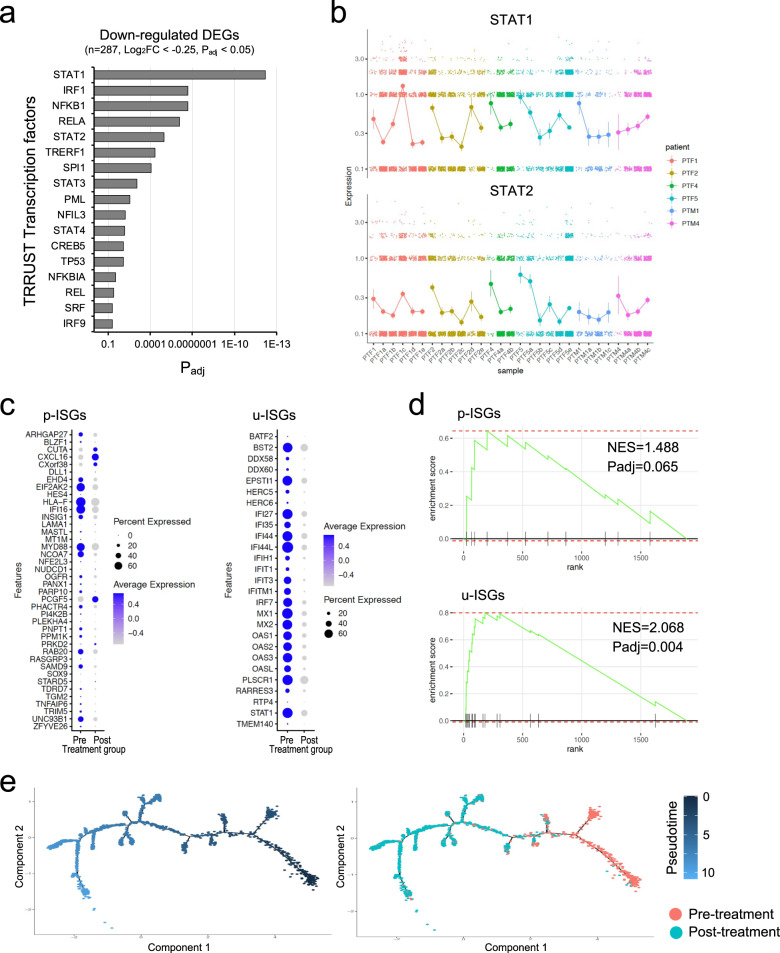
Corticosteroid treatment downregulates the prolonged expression of u-ISGs via STAT1 in classical monocytes (CMs) and intermediate monocytes (IMs). **a** Gene set enrichment analysis of TRRUST transcription factors for genes downregulated by corticosteroid treatment. Padj, adjusted *P* value. **b** Expression of STAT1 and STAT2 in longitudinal samples obtained during the course of corticosteroid treatment. Colors represent individual patients. Error bars indicate the SD. **c** Dot plots showing p-ISGs and u-ISGs changed between pre- and post-corticosteroid treatment. Dot size indicates the percentage of cells in which the gene was detected (“Percent Expressed”). Color indicates the average expression level of the gene (scaled data) in each cluster (“Average Expression”). **d** Gene set enrichment analysis of p-ISGs and u-ISGs changed between pre- and post-corticosteroid treatment. NES, normalized enrichment score; Padj, adjusted *P* value. **e** Trajectory plots for CMs/IMs from samples collected pre- and post-corticosteroid treatment. Cells were ordered by the expression of u-ISGs. Colors represent pseudotime (left) or samples (right).

We also analyzed transcription factors in CMs/IMs potentially related to the effects of corticosteroid treatment. By gene set enrichment analysis with TRRUST transcription factors, STAT1-dependent genes were found to be significantly downregulated by corticosteroid treatment (Fig. 4a), confirming the downregulation of type I IFN-responsive genes by corticosteroid treatment. IRF1-dependent genes were also significantly downregulated by corticosteroid treatment. IRF1 is a transcription factor that can be upregulated by type I IFNs, and its downstream genes contribute to inflammatory responses^35^. This finding supports the downregulation of type I IFN-related pathways being involved in the anti-inflammatory effects of corticosteroids.

Because type I IFN-responsive genes, also known as IFN-stimulated genes (ISGs), were downregulated by corticosteroid treatment, we examined the kinetics of ISG expression in longitudinal samples obtained during the course of corticosteroid treatment. We found that the expression of some ISGs (*IFI27*, *IFI44*, *MX1*, and *OAS3*) was markedly decreased in CMs/IMs after corticosteroid treatment, whereas the expression of another set of ISGs (*EHD4*, *IFI16*, *MYD88*, and *UNC93B1*) was not changed even after corticosteroid treatment (Supplementary Fig. 6a, b). Thus, corticosteroid treatment downregulates the expression of a subset of ISGs, not all ISGs.

Next, we analyzed the expression of STAT1 and two different classes of ISGs, ISGs regulated by unphosphorylated ISGF3 (u-ISGs) and ISGs regulated by phosphorylated ISGF3 (p-ISGs) (Supplementary Fig. 7)^36,37^. STAT1 expression was significantly downregulated in CMs/IMs obtained 1 day after corticosteroid treatment (Fig. 4b). Although multiple u-ISGs in CMs/IMs were downregulated by corticosteroid treatment, the modulation of p-ISGs was less prominent (Fig. 4c). By gene set enrichment analysis, u-ISGs were found to be significantly enriched in genes downregulated by corticosteroids, whereas p-ISGs were not (Fig. 4d). We also performed semisupervised pseudotime trajectory analysis by ordering cells using the u-ISGs; pre- and post-treatment cells were clearly segregated along the pseudotime axis (Fig. 4e and Supplementary Fig. 8a), suggesting that u-ISGs can be molecular hallmarks of corticosteroid responses. We found a drastic downregulation pattern for u-ISGs, including IFI27, IFI44, MX1, and OAS3, after corticosteroid treatment (Supplementary Fig. 8b). Taken together, the results suggest that the anti-inflammatory effects of corticosteroids are related to the downregulation of STAT1 and STAT1-dependent u-ISGs.

### Enrichment of u-ISGs downregulated by corticosteroids in monocytes from patients with lupus

**Fig. 5:**
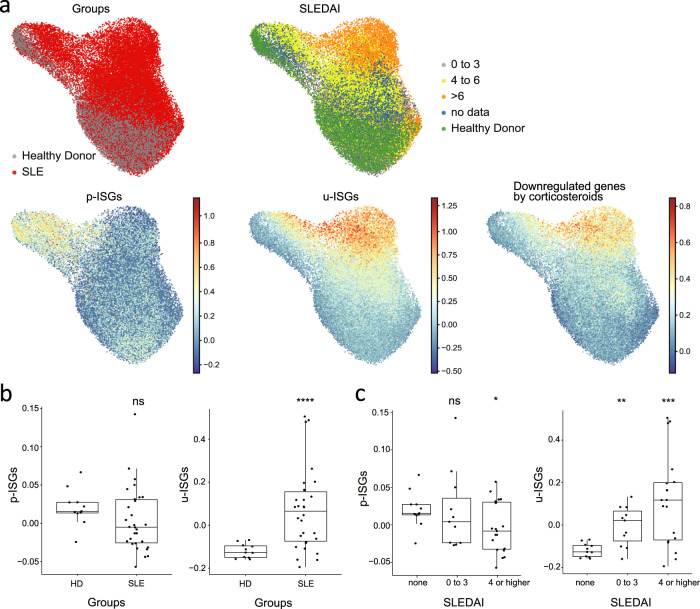
Transcriptomic features of monocytes from lupus patients are associated with u-ISGs and genes downregulated by corticosteroid treatment. **a** UMAP plot of monocyte subclusters originating from publicly available single-cell transcriptome data^36^ showing the expression patterns of p-ISGs, u-ISGs and downregulated genes after corticosteroid treatment. **b** Box-and-whisker plots of the different expression levels of p-ISGs and u-ISGs between healthy donors and lupus patients. **c** Box-and-whisker plots of the different expression levels of p-ISGs and u-ISGs among healthy donors (none) and patients with an SLE disease activity index (SLEDAI) of 0 to 3 or ≥4. The box of each boxplot starts in the first quartile and ends in the third, with the line inside representing the median.

We questioned whether u-ISGs downregulated by corticosteroid treatment in patients with high-risk COVID-19 are involved in other diseases. First, we performed KEGG pathway analysis using u-ISGs (*n* = 29). Eight of the top 10 enriched pathways were viral infection-related pathways (red bar, Supplementary Fig. 8c), confirming the enrichment of u-ISGs in various viral diseases. Next, we analyzed whether u-ISGs are enriched in other inflammatory diseases in which type I IFNs play a pathological role. To this end, we examined the expression of u-ISGs in peripheral blood monocytes from patients with systemic lupus erythematosus (SLE) and healthy controls using publicly available scRNA-seq data^38^. Among monocytes, the expression of u-ISG was preferentially upregulated in cells enriched in SLE patients with a high SLE disease activity index (SLEDAI) score, whereas the expression of p-ISGs was not (Fig. 5a). Thus, u-ISGs were strongly associated with hyperinflammation in patients with SLE. In addition, genes downregulated by corticosteroid treatment in patients with high-risk COVID-19 were preferentially upregulated in cells enriched in SLE patients with high SLEDAI scores. By statistical analysis, we confirmed that the expression of u-ISGs was significantly increased in monocytes from SLE patients compared to those from healthy controls, but the expression of p-ISGs was not (Fig. 5b). Moreover, the expression of u-ISGs was significantly increased in monocytes from SLE patients with higher SLEDAI scores compared to those from patients with lower SLEDAI scores, but the expression of p-ISGs was not (Fig. 5c). Taken together, the results indicate that u-ISGs that were identified as corticosteroid-downmodulated genes in patients with high-risk COVID-19 are highly enriched in SLE patients with higher disease activity; thus, u-ISGs are involved in hyperinflammation during the pathogenesis of high-risk COVID-19.

### Shared features between corticosteroid treatment and spontaneous improvement

**Fig. 6:**
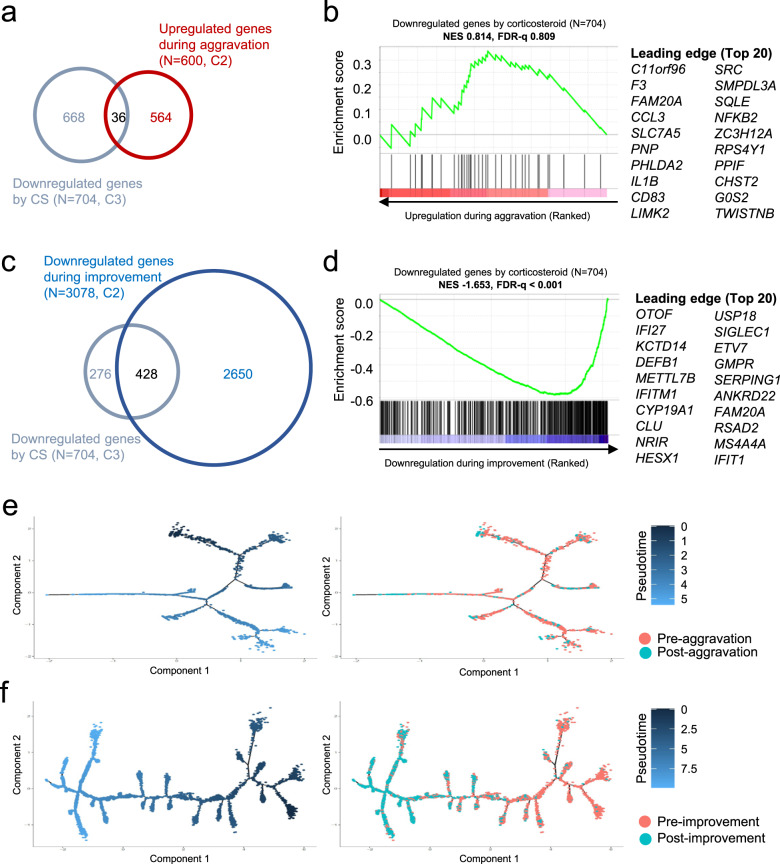
Similarities between genes downregulated by corticosteroid (CS) treatment and genes with altered expression during the course of COVID-19 in classical monocytes (CMs) and intermediate monocytes (IMs). **a** Venn diagram showing the overlap of genes downregulated by CS treatment (*n* = 704, C3) and genes upregulated during aggravation of COVID-19 (*n* = 600, C2). **b** Gene set enrichment analysis of genes upregulated during aggravation of COVID-19 (*n* = 600, C2 in **a**) for gene sets downregulated by CS treatment. FDR, false discovery rate; NES, normalized enrichment score. **c** Venn diagram showing the overlap of genes downregulated by CS treatment (*n* = 704, C3) and genes downregulated during improvement of COVID-19 (*n* = 3078, C2). **d** Gene set enrichment analysis of genes downregulated during the improvement of COVID-19 (*n* = 3078, C2 in **c**) for gene sets downregulated by CS treatment. **e**, **f** Trajectory plots for CMs/IMs in samples from cohort 2 during disease aggravation (**e**) or improvement (**f**). The cells were ordered by the expression of u-ISGs. Colors represent pseudotime (left) or samples (right).

Finally, we investigated whether corticosteroid treatment-induced changes were observed during COVID-19 aggravation or improvement. To this end, we analyzed downregulated genes in CMs/IMs after corticosteroid treatment compared to DEGs retrieved from cohort 2. The genes downregulated by corticosteroid treatment did not overlap much with the genes upregulated during disease aggravation and were not enriched with any genes that were upregulated during disease aggravation (Fig. 6a, b). However, the genes downregulated by corticosteroid treatment considerably overlapped with the genes downregulated during disease improvement and were significantly enriched with the genes that were downregulated during disease improvement (Fig. 6c, d). We also performed semisupervised pseudotime trajectory analysis using u-ISGs during disease aggravation and improvement. Although we found no obvious correlation between disease aggravation and the pseudotime axis, cells from the improvement course were successfully ordered by the pseudotime axis (Fig. 6e, f). Therefore, the downregulation of u-ISGs that was observed during corticosteroid treatment also occurred during spontaneous improvement during COVID-19, suggesting that the anti-inflammatory process induced by corticosteroid treatment resembles the spontaneous resolution process in COVID-19.

## Discussion

Progression to severe COVID-19 is caused by hyperinflammatory responses characterized by exaggerated activation of myeloid cells, such as neutrophils and monocytes/macrophages, and the overproduction of proinflammatory cytokines, such as TNF, IL-6, and IL-1β^6–9,12^. Since early after the emergence of SARS-CoV-2, corticosteroids have been used for the treatment of patients with high-risk COVID-19 and have shown clinical benefits to patients with high-risk COVID-19^21^. However, we do not yet understand exactly how corticosteroids attenuate hyperinflammatory responses and alleviate high-risk COVID-19.

In the current study, we examined the effects of corticosteroid treatment on peripheral blood immune cells from patients with high-risk COVID-19 by performing scRNA-seq analysis. We obtained key results from paired PBMC samples obtained immediately before and 1 day after starting corticosteroid treatment. We first found that CMs/IMs underwent the most striking changes after corticosteroid treatment in terms of the number of DEGs. In CMs/IMs, the mTOR and TGFB pathways were upregulated, and the type I IFN and MHC/adhesion pathways were downregulated after corticosteroid treatment. In further analyses, corticosteroid treatment downregulated the expression of *STAT1* in CMs. In addition, corticosteroid-induced downregulation of type I IFN-responsive genes was observed selectively among u-ISGs that are known to depend on prolonged high levels of STAT1.

In COVID-19, early robust type I IFN responses are observed in patients with mild disease^17,20^, whereas type I IFN deficiency leads to progression to severe COVID-19 due to insufficient antiviral activity^18,19^. However, paradoxical inflammatory roles for type I IFNs have been reported in patients with severe COVID-19, particularly delayed but exaggerated type I IFN responses in monocytes^11,20,31,39,40^. In mouse models, monocytes affected by delayed and exaggerated type I IFN responses lead to lethal or severe SARS or COVID-19^39,41^. Hyperinflammatory roles for type I IFNs have been reported in other models of inflammation^42,43^. In particular, abrogation of TNF-induced TLR tolerance by type I IFNs has been shown to be involved in the hyperinflammatory responses of monocytes^44^. In this context, downregulation of type I IFN responses may be a major mechanism by which corticosteroids alleviate high-risk COVID-19 and provide clinical benefits to patients.

Canonical signaling by type I IFNs relies on the phosphorylation of STATs, and ISGF3, a trimer of p-STAT1, p-STAT2, and IRF9, induces the expression of various ISGs^45^. However, noncanonical signaling has been demonstrated. It depends on high levels of STAT1, STAT2, and IRF9 rather than phosphorylation of STATs^35,46^. Prolonged type I IFN stimulation increases the expression levels of STAT1, STAT2, and IRF9, and upregulated STAT1, STAT2, and IRF9 can form u-ISGF3 even without STAT phosphorylation to induce the expression of u-ISGs^36,37^. Interestingly, in the current study, corticosteroid treatment immediately downregulated the expression of *STAT1* in CMs/IMs. In line with this finding, corticosteroid treatment selectively downregulated the expression of u-ISGs that directly depend on high levels of STAT1. Interestingly, high levels of u-ISGs were found in monocytes from SLE patients with particularly high disease activity. In addition, genes downregulated by corticosteroids were also highly enriched in monocytes from SLE patients with particularly high disease activity. Given that SLE is a representative inflammatory disease that involves pathological type I IFN activity^47^, our current data strongly suggest that u-ISGs directly contribute to hyperinflammation in patients with high-risk COVID-19 and are targeted by corticosteroid treatment.

COVID-19 spontaneously resolves in almost all patients, particularly in children and young adults^3^. However, which molecular events occur during spontaneous improvement of COVID-19 has not been studied, although the mechanisms underlying progression to severe COVID-19 have been studied extensively. Given that there are concerns about long COVID or post-acute COVID-19 syndrome^4,48^, it is important to understand the process of COVID-19 resolution. In the current study, we examined transcriptomic changes during the course of COVID-19 improvement. In the cell-to-cell interactome analysis, multiple genes involved in angiogenesis and wound healing, including *CXCL1, CXCL2, CXCL3, CXCR2, VEGFA*, and *HBEGF*, were upregulated in CMs/IMs during improvement. It is necessary to examine whether dysregulated angiogenesis and wound healing responses are involved in incomplete recovery from COVID-19 and long COVID.

Although they are used to treat patients with high-risk COVID-19, corticosteroids need to be carefully administered to patients because of their immunosuppressive effects. The recent high incidence of mucormycosis, a rare fungal infection, is thought to be associated with the wide use of corticosteroids^49^. In the current study, we found that corticosteroid treatment downregulated multiple MHC genes in CMs/IMs, indicating that corticosteroids attenuate the antigen-presenting capability of monocytes. Antigen presentation is the first step in the priming and activation of CD4^+^ and CD8^+^ T cells, which play critical roles in viral control^50^. In patients with high-risk COVID-19, MHC and antigen presentation-related genes are downregulated, impairing the activation of CD4^+^ and CD8^+^ T cells^5,33^. In these patients, corticosteroid treatment further reduces the expression of MHC and antigen presentation-related genes, exacerbating immunosuppression and leading to secondary infections in extreme cases.

In the current study, we investigated the immunological dynamics of COVID-19 by performing scRNA-seq analysis. Our results showed transcriptomic changes in diverse types of immune cells during disease aggravation and improvement. In addition, our results revealed the effects of corticosteroid treatment at the transcriptomic level and showed that corticosteroid treatment normalized the lupus-like dysregulated type I IFN response. The current study provides knowledge to improve the understanding the inflammatory response and its modulation in COVID-19, allowing the development of more selective anti-inflammatory agents with fewer immunosuppressive effects for the treatment of high-risk COVID-19.

## Supplementary information

Supplementary Figures, Tables, and Legends

Supplementary table 1

Supplementary table 2

## Data Availability

The demultiplexed raw gene expression data in this paper were deposited in the NCBI GEO database under the accession number GSE188172. The experimental protocols and data analysis pipeline used in our work followed the instructions of BD Biosciences and the vignettes of the Seurat official website. The analysis steps, functions, and parameters used are described in detail in the Methods section. Custom scripts for data analysis are available upon reasonable request.

## References

[1] Wu F (2020). A new coronavirus associated with human respiratory disease in China. Nature.

[2] Zhu N (2020). A novel coronavirus from patients with pneumonia in China, 2019. N. Engl. J. Med..

[3] Long QX (2020). Clinical and immunological assessment of asymptomatic SARS-CoV-2 infections. Nat. Med..

[4] Huang C (2020). Clinical features of patients infected with 2019 novel coronavirus in Wuhan, China. Lancet.

[5] Schulte-Schrepping J (2020). Severe COVID-19 is marked by a dysregulated myeloid cell compartment. Cell.

[6] Merad M, Martin JC (2020). Pathological inflammation in patients with COVID-19: a key role for monocytes and macrophages. Nat. Rev. Immunol..

[7] Xu G (2020). The differential immune responses to COVID-19 in peripheral and lung revealed by single-cell RNA sequencing. Cell Discov..

[8] Kuri-Cervantes L (2020). Comprehensive mapping of immune perturbations associated with severe COVID-19. Sci. Immunol..

[9] Del Valle DM (2020). An inflammatory cytokine signature predicts COVID-19 severity and survival. Nat. Med..

[10] Ma L (2021). Increased complement activation is a distinctive feature of severe SARS-CoV-2 infection. Sci. Immunol..

[11] Lee JS, Shin EC (2020). The type I interferon response in COVID-19: implications for treatment. Nat. Rev. Immunol..

[12] Schultze JL, Aschenbrenner AC (2021). COVID-19 and the human innate immune system. Cell.

[13] Ren X (2021). COVID-19 immune features revealed by a large-scale single-cell transcriptome atlas. Cell.

[14] Wilk AJ (2020). A single-cell atlas of the peripheral immune response in patients with severe COVID-19. Nat. Med..

[15] Liao M (2020). Single-cell landscape of bronchoalveolar immune cells in patients with COVID-19. Nat. Med..

[16] Lee JS (2020). Immunophenotyping of COVID-19 and influenza highlights the role of type I interferons in development of severe COVID-19. Sci. Immunol..

[17] Kim YM, Shin EC (2021). Type I and III interferon responses in SARS-CoV-2 infection. Exp. Mol. Med..

[18] Zhang Q (2020). Inborn errors of type I IFN immunity in patients with life-threatening COVID-19. Science.

[19] Bastard P (2020). Autoantibodies against type I IFNs in patients with life-threatening COVID-19. Science.

[20] Carvalho T, Krammer F, Iwasaki A (2021). The first 12 months of COVID-19: a timeline of immunological insights. Nat. Rev. Immunol..

[21] Group RC (2021). Dexamethasone in Hospitalized Patients with Covid-19. N. Engl. J. Med..

[22] Kirwan JR (1995). The effect of glucocorticoids on joint destruction in rheumatoid arthritis. The Arthritis and Rheumatism Council Low-Dose Glucocorticoid Study Group. N. Engl. J. Med..

[23] Hench PS, Kendall EC, Slocumb CH, Polley HF (1949). The effect of a hormone of the adrenal cortex (17-hydroxy-11-dehydrocorticosterone: compound E) and of pituitary adrenocortical hormone in arthritis: preliminary report. Ann. Rheum. Dis..

[24] Schauer M, Chalepakis G, Willmann T, Beato M (1989). Binding of hormone accelerates the kinetics of glucocorticoid and progesterone receptor binding to DNA. Proc. Natl Acad. Sci. USA.

[25] Oh KS (2017). Anti-inflammatory chromatinscape suggests alternative mechanisms of glucocorticoid receptor action. Immunity.

[26] Jick SS, Lieberman ES, Rahman MU, Choi HK (2006). Glucocorticoid use, other associated factors, and the risk of tuberculosis. Arthritis Rheum..

[27] Park JW (2018). Prophylactic effect of trimethoprim-sulfamethoxazole for pneumocystis pneumonia in patients with rheumatic diseases exposed to prolonged high-dose glucocorticoids. Ann. Rheum. Dis..

[28] Liberzon A (2015). The Molecular Signatures Database (MSigDB) hallmark gene set collection. Cell Syst..

[29] Smith GB, Prytherch DR, Meredith P, Schmidt PE, Featherstone PI (2013). The ability of the National Early Warning Score (NEWS) to discriminate patients at risk of early cardiac arrest, unanticipated intensive care unit admission, and death. Resuscitation.

[30] Romagnani P, Lasagni L, Annunziato F, Serio M, Romagnani S (2004). CXC chemokines: the regulatory link between inflammation and angiogenesis. Trends Immunol..

[31] Rodrigues SF, Granger DN (2015). Blood cells and endothelial barrier function. Tissue Barriers.

[32] Weichhart T (2011). Inhibition of mTOR blocks the anti-inflammatory effects of glucocorticoids in myeloid immune cells. Blood.

[33] Lucas C (2020). Longitudinal analyses reveal immunological misfiring in severe COVID-19. Nature.

[34] Giamarellos-Bourboulis EJ (2020). Complex immune dysregulation in COVID-19 patients with severe respiratory failure. Cell Host Microbe.

[35] Forero A (2019). Differential activation of the transcription factor IRF1 underlies the distinct immune responses elicited by type I and type III interferons. Immunity.

[36] Cheon H (2013). IFNbeta-dependent increases in STAT1, STAT2, and IRF9 mediate resistance to viruses and DNA damage. EMBO J..

[37] Sung PS (2015). Roles of unphosphorylated ISGF3 in HCV infection and interferon responsiveness. Proc. Natl Acad. Sci. USA.

[38] Nehar-Belaid D (2020). Mapping systemic lupus erythematosus heterogeneity at the single-cell level. Nat. Immunol..

[39] Sariol A, Perlman S (2020). Lessons for COVID-19 immunity from other coronavirus infections. Immunity.

[40] Channappanavar R (2016). Dysregulated type I interferon and inflammatory monocyte-macrophage responses cause lethal pneumonia in SARS-CoV-infected mice. Cell Host Microbe.

[41] Israelow B (2020). Mouse model of SARS-CoV-2 reveals inflammatory role of type I interferon signaling. J. Exp. Med..

[42] Gonzalez-Navajas JM, Lee J, David M, Raz E (2012). Immunomodulatory functions of type I interferons. Nat. Rev. Immunol..

[43] McNab F, Mayer-Barber K, Sher A, Wack A, O’Garra A (2015). Type I interferons in infectious disease. Nat. Rev. Immunol..

[44] Park SH (2017). Type I interferons and the cytokine TNF cooperatively reprogram the macrophage epigenome to promote inflammatory activation. Nat. Immunol..

[45] Platanias LC (2005). Mechanisms of type-I- and type-II-interferon-mediated signalling. Nat. Rev. Immunol..

[46] Majoros A (2016). Response to interferons and antibacterial innate immunity in the absence of tyrosine-phosphorylated STAT1. EMBO Rep..

[47] Morand EF (2020). Trial of anifrolumab in active systemic Lupus Erythematosus. N. Engl. J. Med..

[48] Nalbandian A (2021). Post-acute COVID-19 syndrome. Nat. Med..

[49] Mahalaxmi I (2021). Mucormycosis: an opportunistic pathogen during COVID-19. Environ. Res..

[50] Rha MS, Kim AR, Shin EC (2021). SARS-CoV-2-specific T cell responses in patients with COVID-19 and unexposed individuals. Immune Netw..

