# Supplementary material for: Corticosteroids reduce pathologic interferon responses by downregulating STAT1 in patients with high-risk COVID-19
Source: Exp Mol Med. 2023 Mar 20;55(3):653–64. doi: 10.1038/s12276-023-00964-8 (PMC10026241; doi:10.1038/s12276-023-00964-8)
Supplement: Supplementary file 1 — Supplementary Figures, Tables, and Legends [file 12276_2023_964_MOESM1_ESM.pdf]

Supplementary Figure 1

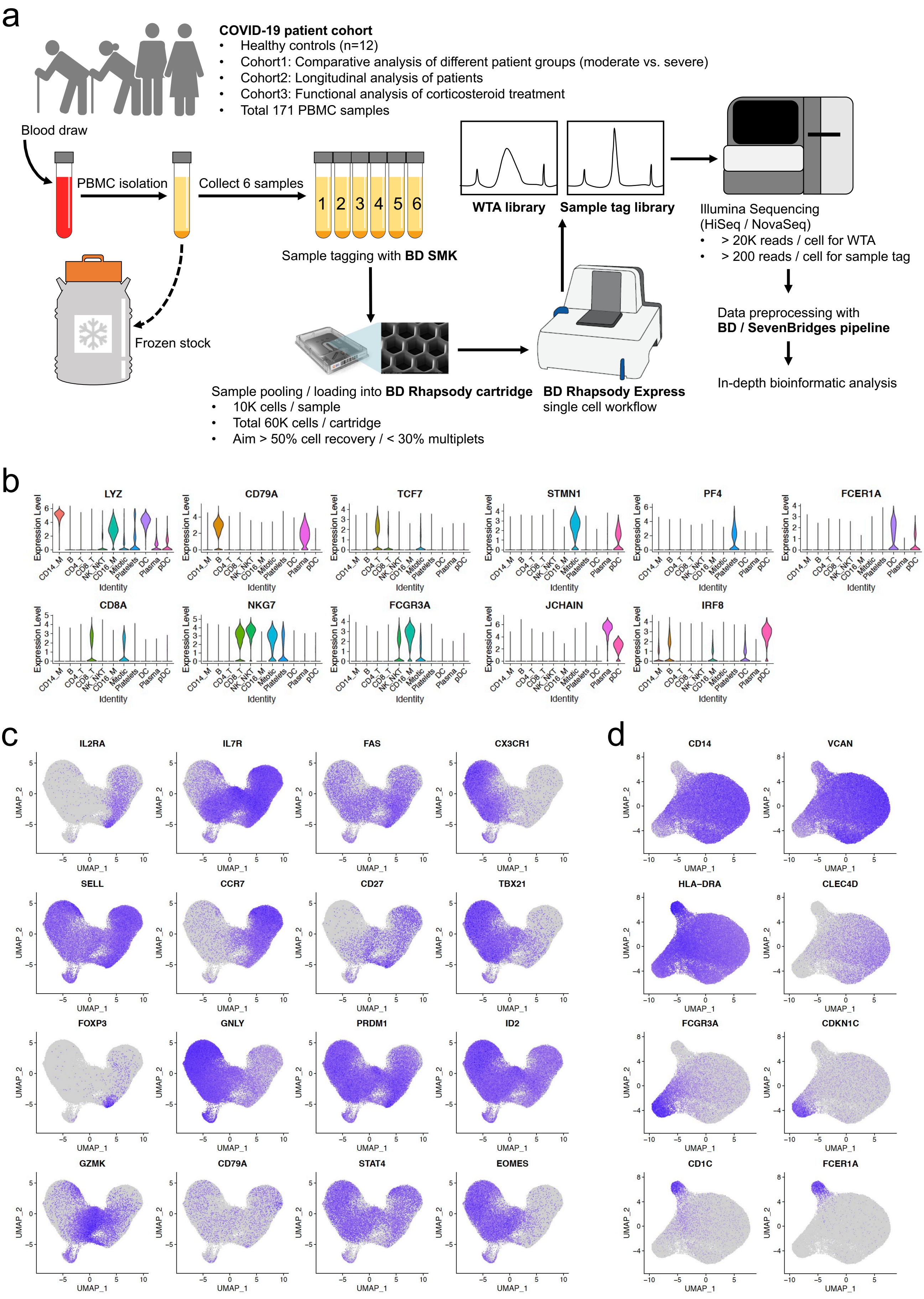

Supplementary Figure 1. Workflow of the analysis of single-cell RNA-seq and expression of cell-type-specific marker genes. (**a**) Schematic overview of single-cell RNA-seq using PBMCs. (**b**) Expression of select cell-type specific marker genes in major cell types in violin plots and (**c**) lymphoid or (**d**) myeloid cell subpopulations in UMAP plots. (c,d) Colors represent the relative expression of each gene.

Supplementary Figure 2

a

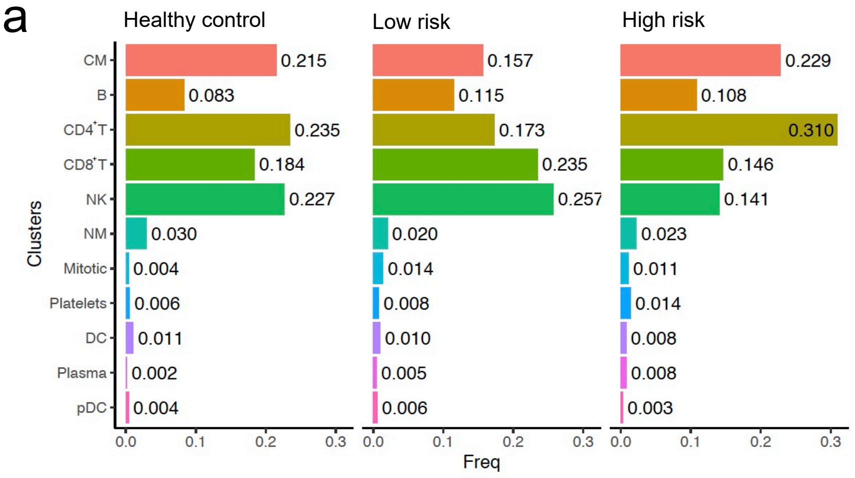

b

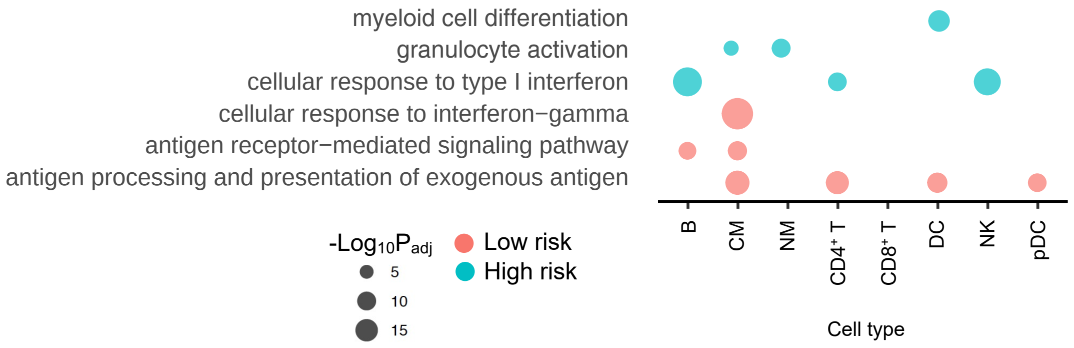

Supplementary Figure 2. Comparison of samples with different disease severity. (**a**) Cell type frequency of each group. (**b**) Six biological process Gene Ontology terms enriched in high risk or low risk patients' samples in each cell type. Dot size represents -Log10 (adjusted P-value).

Supplementary Figure 3

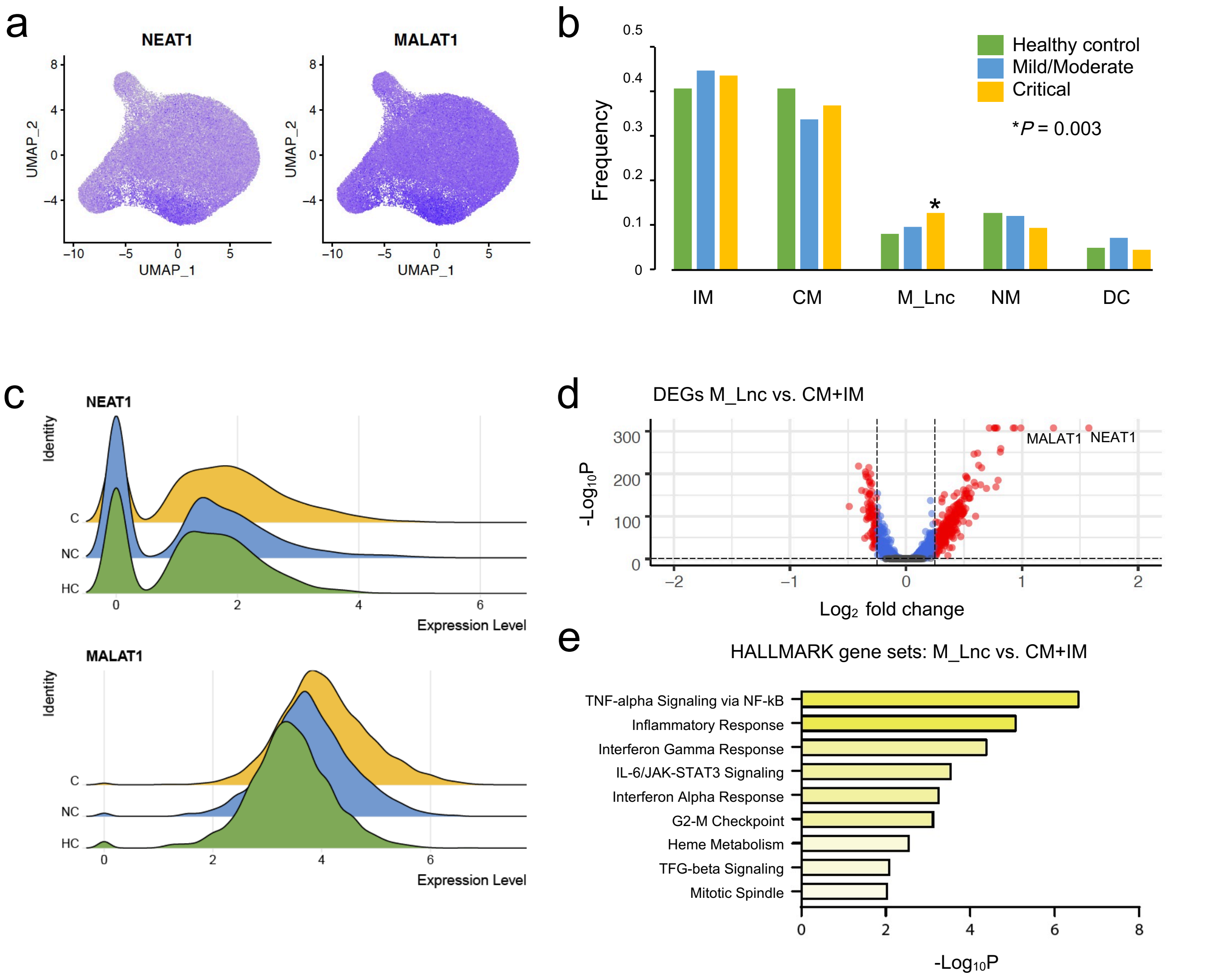

Supplementary Figure 3. Hyperinflammatory hallmarks of myeloid cells in high risk COVID-19 patients. (**a**) UMAP plots depicting the expression of NEAT1 and MALAT1 in the myeloid cell population. Colors represent relative gene expression. (**b**) Bar plot showing the frequency of myeloid subtypes in different patient groups. (**c**) Ridge plots depicting the expression of NEAT1 and MALAT1 in different patient groups. (**d**) Volcano plot of differentially expressed genes between M_Lnc and CM + IM. Blue dots, adjusted P < 0.05; Red dots, adjusted P < 0.05 and Log2 fold change > 0.25; grey dots, adjusted P > 0.05. (**e**) Top enriched HALLMARK gene sets in M_Lnc.

Supplementary Figure 4

a

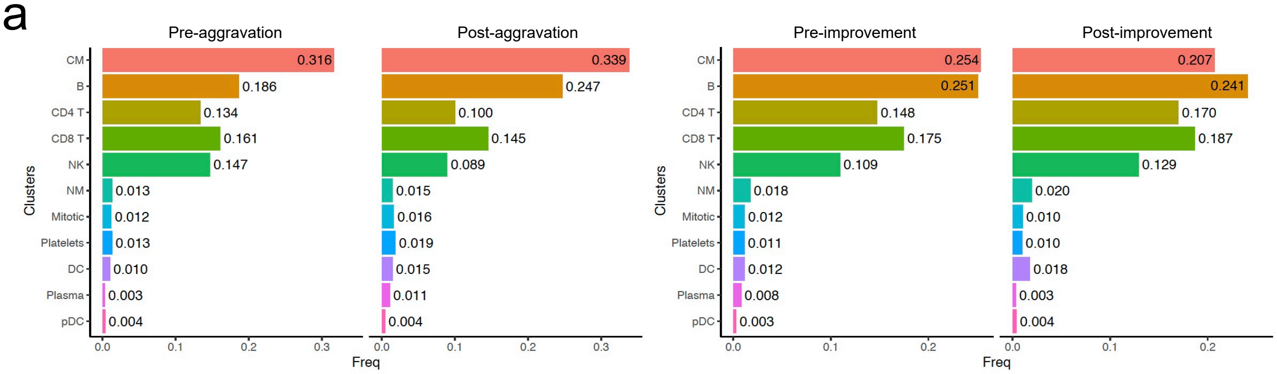

b

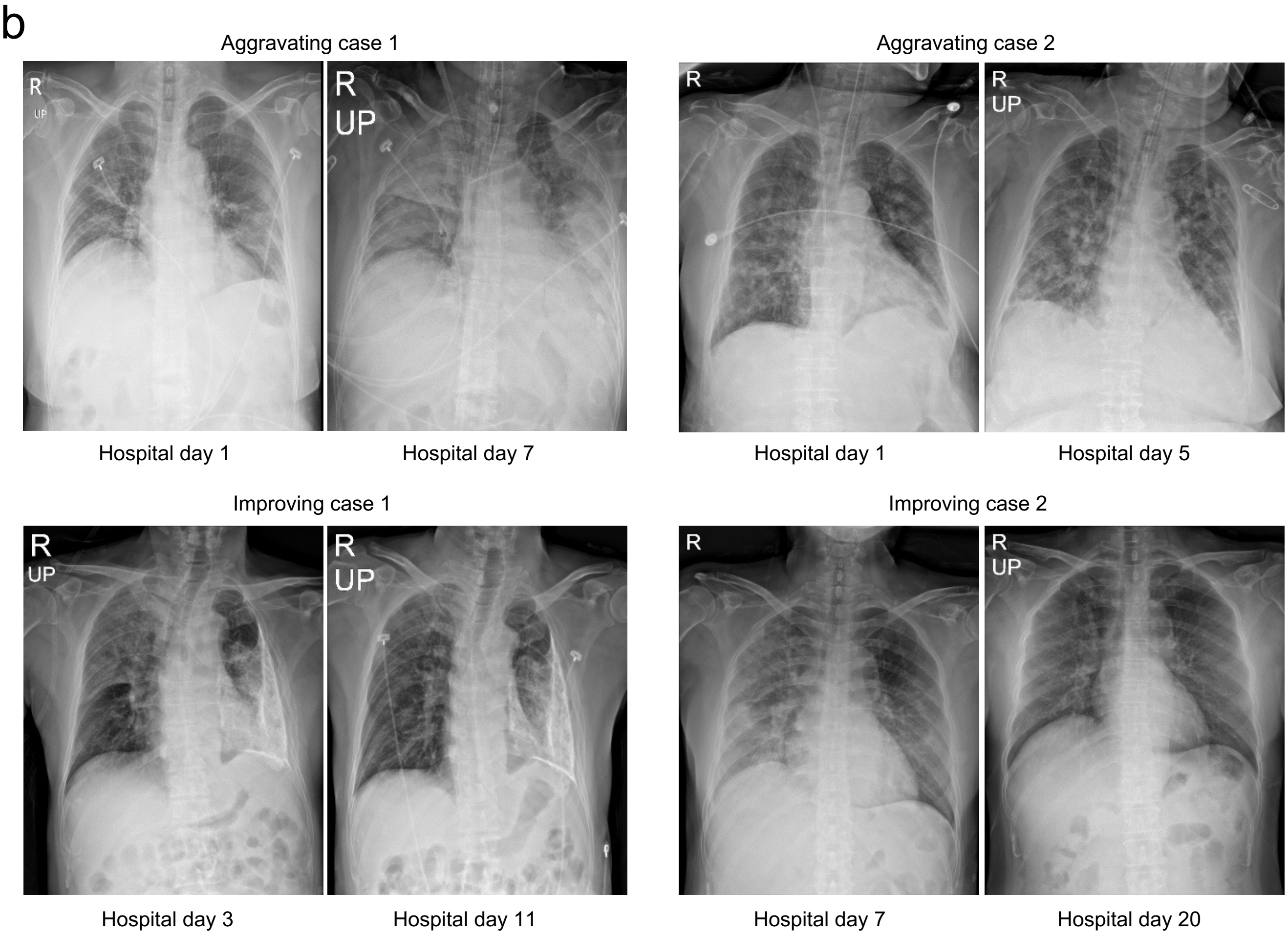

c

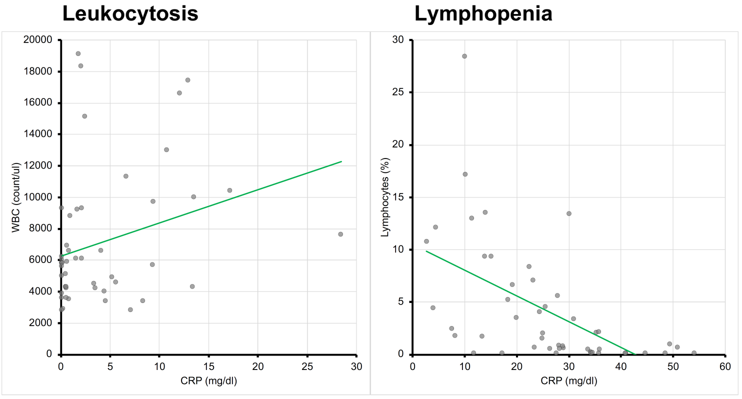

d

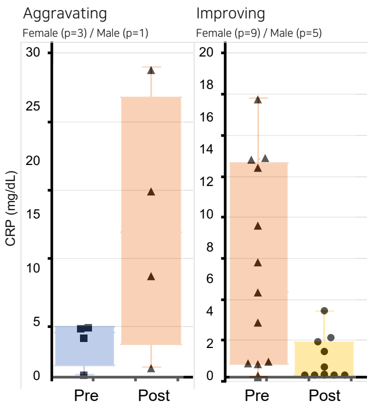

Supplementary Figure 4. Phenotypic changes during COVID-19 progression. (**a**) Cell type frequency of during disease aggravation and improvement. (**b**) Chest radiographs of representative cases of disease aggravation (top) and improvement (bottom). The day after hospitalization is shown for each patient. (**c**) Relationship of whole white blood cell (WBC) counts and lymphocyte frequency (%) with serum c-reactive protein (CRP) levels (mg/dl). Each dot represents a sample. (**d**) Box-and-whisker plots of CRP levels in select patient samples based on disease aggravation and improvement. The box of a boxplot starts in the first quartile and ends in the third, with a line inside that represents the median.

Supplementary Figure 5

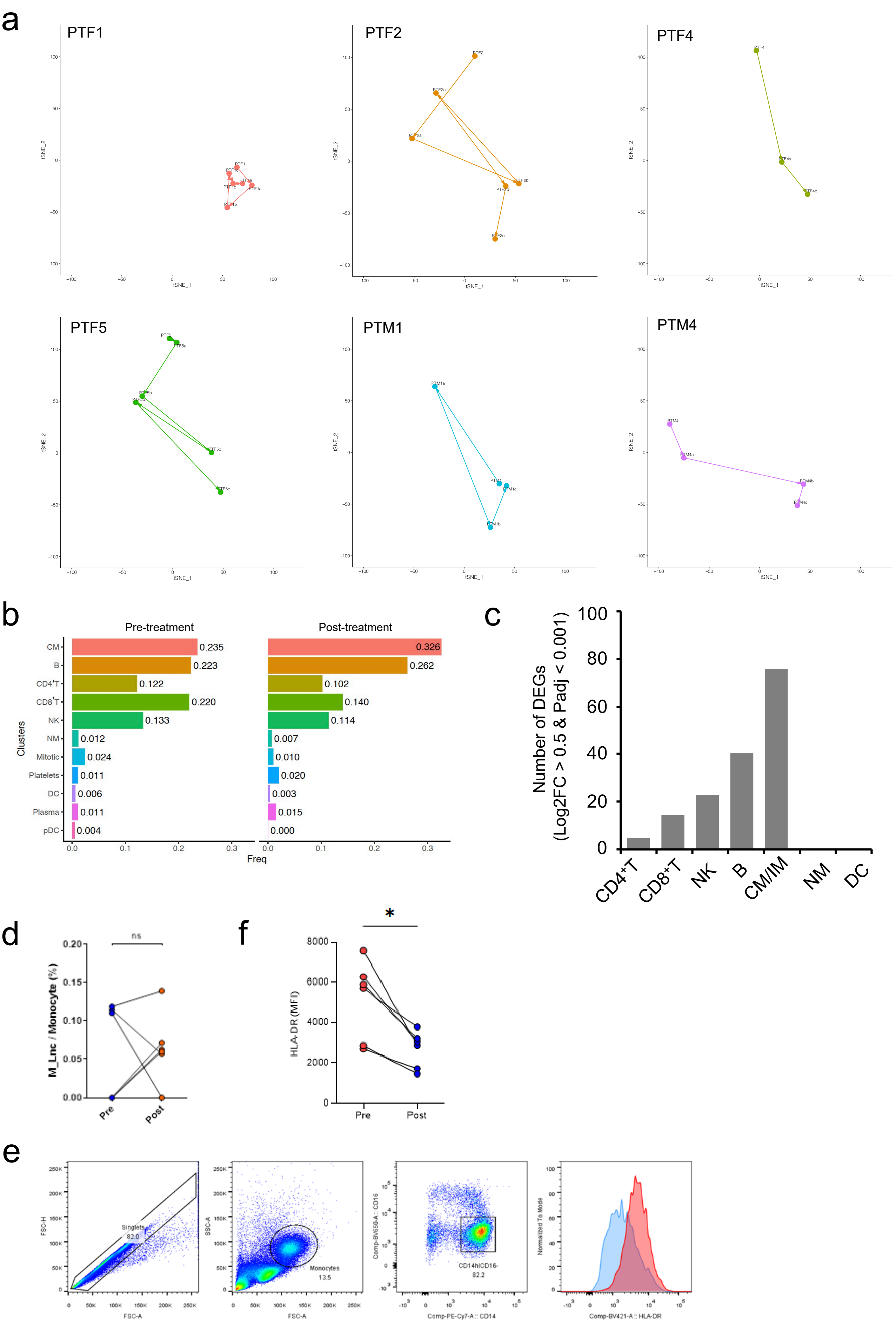

Supplementary Figure 5. Corticosteroid-induced changes in gene expression in PBMCs. (**a**) tSNE plot of pseudo-bulk gene expression values for CMs/IMs in each sample. Colors represent individual patients. Arrows indicate progression of time. Six patients with pre-treated conditions are labeled with text. (**b**) Cell type frequency in PBMCs from corticosteroid-treated patients pre- and post-treatment. PTM1, a patient with acute leukemia, was excluded from this analysis. (**c**) Number of significant differentially expressed genes (DEGs) in each cell type after corticosteroid treatment. (**d**) Frequency of monocyte_Lnc among myeloid subclusters. (**e**) Representative dot blot presenting cell surface HLA-DR expression level of $CD14^{+}CD16^{-}$ classical monocytes. (**f**) HLA-DR surface expression level of $CD14^{+}CD16^{-}$ classical monocytes of pre- and post-CS treatment

Supplementary Figure 6

a

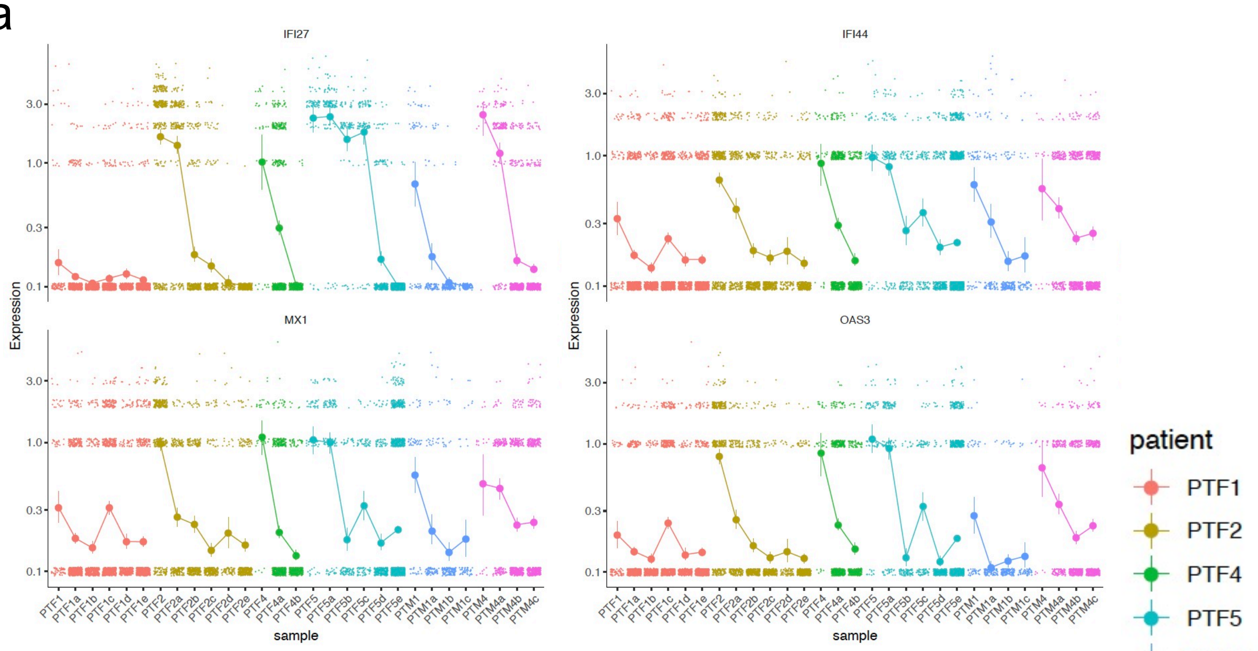

b

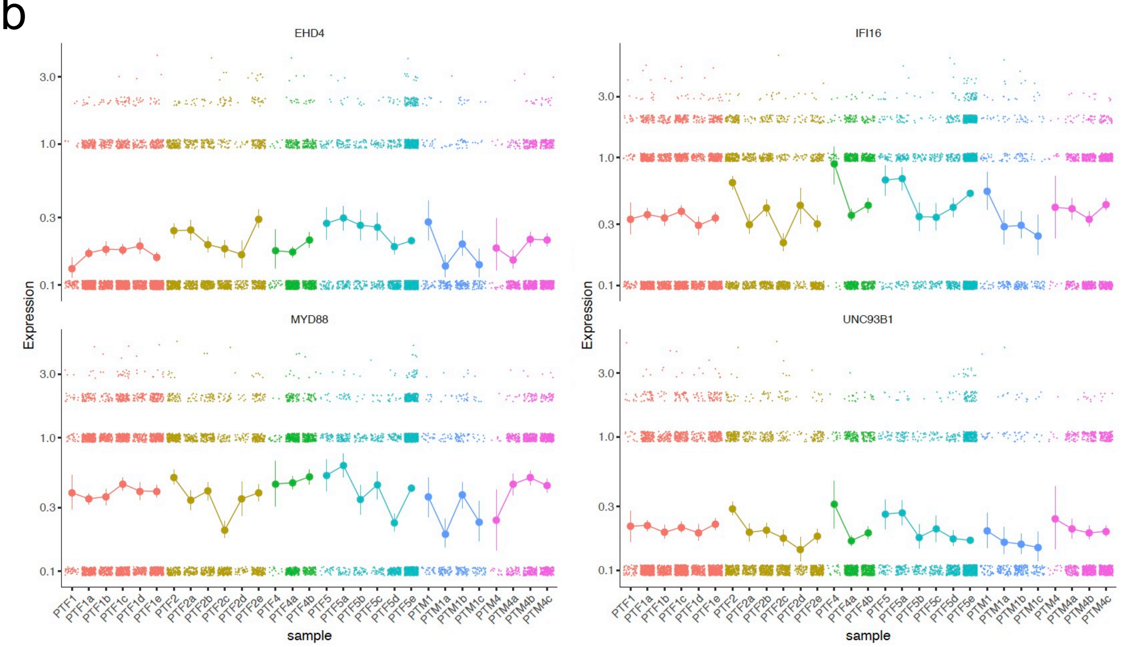

Supplementary Figure 6. Corticosteroid treatment suppresses a subset of u-ISG expression. (**a**) Expression of down-regulated or (**b**) unchanged genes in longitudinal samples obtained during the course of corticosteroid treatment. Colors represent individual patients. Error bars indicate SD.

Supplementary Figure 7

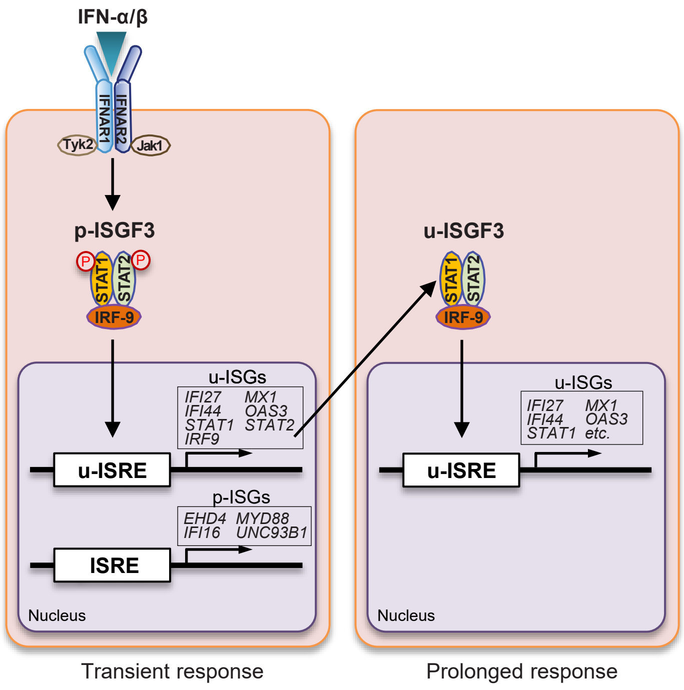

Supplementary Figure 7. Upregulation of p-ISGs and u-ISGs by type I IFNs. Type I IFN stimulation induces phosphorylation of tyrosine residues in STAT1 and STAT2. p-STAT1, p- STAT2, and IRF9 form p-ISGF3, which induces transient expression of p-ISGs by binding to the IFN-sensitive response element (ISRE). Up-regulated STAT1, STAT2, and IRF9 can form u- ISGF3 even without tyrosine phosphorylation, and u-ISGF3 induces prolonged expression of u-ISGs by binding to u-ISRE3.

Supplementary Figure 8

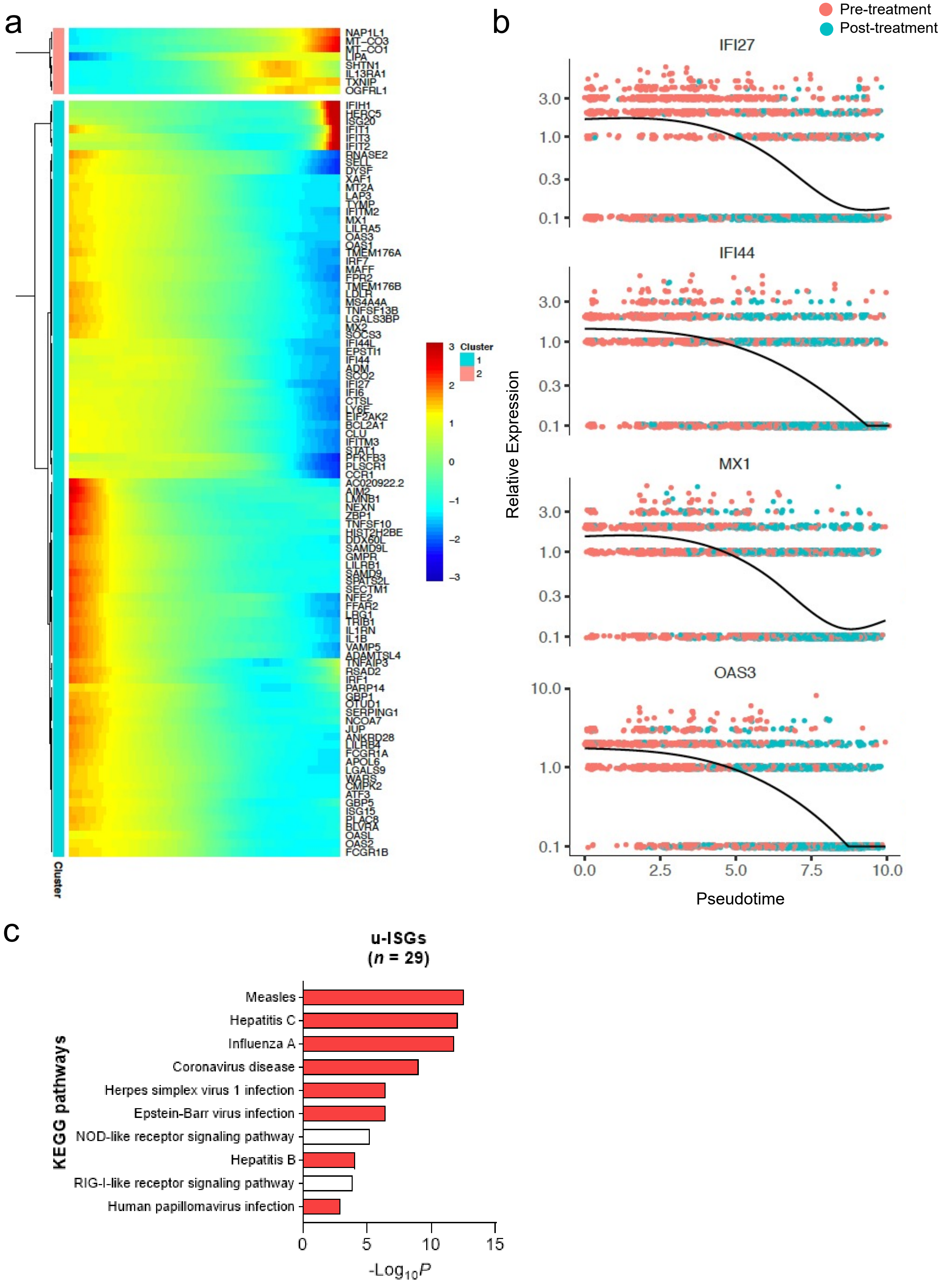

Supplementary Figure 8. Pseudotime analysis of classical monocytes/intermediate monocytes with u-ISGs. (**a**) Heatmap of differentially expressed genes by pseudotime. Cells were ordered by the expression of u-ISGs. Colors indicate the relative expression of each gene. (**b**) Expression of selected genes by pseudotime. Colors represent samples of pre- and post-corticosteroid treatment. (**c**) Top 10 enriched KEGG pathways of u-ISGs (n = 29), red bars indicating viral infection associated pathways.
